# Inflammatory profiling and immune cell infiltration in dysthyroid optic neuropathy: insights from bulk RNA sequencing

**DOI:** 10.3389/fimmu.2025.1550694

**Published:** 2025-03-12

**Authors:** Qintao Ma, Yuanping Hai, Yongbo Duan, Genfeng Yu, Cheng Song, ShengAi Huang, Anqi Huang, Yan Zhu, Yongzhi Shen, Zimeng Huang, Xiao Wang, Lan Liu, Thomas Efferth, Huiyu Guo, Yi Wang, Jie Shen

**Affiliations:** ^1^ Department of Endocrinology and Metabolism, Shunde Hospital, Southern Medical University (The First People’s Hospital of Shunde), Foshan, Guangdong, China; ^2^ Department of Ophthalmopathy, Shunde Hospital, Southern Medical University, (The First Peoples’ Hospital of Shunde), Foshan, China; ^3^ Hainan Eye Hospital and Key Laboratory of Ophthalmology, Zhongshan Ophthalmic Center, Sun Yat-sen University, Haikou, Hainan, China; ^4^ Department of Pharmaceutical Biology, Johannes Gutenberg University, Mainz, Germany; ^5^ Department of Ophthalmology, Peking University Third Hospital, Beijing, China; ^6^ Beijing Key Laboratory of Restoration of Damaged Ocular Nerve, Peking University Third Hospital, Beijing, China

**Keywords:** dysthyroid optic neuropathy, inflammation-related genes, immune microenvironment, transcriptomic profiling, fibrosis, thyroid eye disease

## Abstract

**Background:**

Dysthyroid optic neuropathy (DON), the most severe complication of thyroid eye disease (TED), has unclear mechanisms and unsatisfactory treatment outcomes. This study aimed to identify key pathways and inflammation-related core genes driving DON progression, potentially informing new therapeutic strategies and improving disease management.

**Methods:**

Retro-orbital tissues from DON, non-DON TED, and healthy controls (HCs) were analyzed using bulk RNA sequencing. Differentially expressed genes (DEGs) were identified and subjected to Gene Ontology (GO) enrichment analysis. Weighted gene co-expression network analysis (WGCNA) identified disease-relevant modules. Immune cell infiltration was assessed via single-sample Gene Set Enrichment Analysis (ssGSEA). ROC analysis and single-gene GSEA were used to evaluate the diagnostic potential and functional relevance of core genes. Inflammatory-Related Differential Genes (IRDGS) were identified and preliminarily validated using Quantitative Real-Time PCR.

**Results:**

Differential gene expression analysis revealed 176 and 202 significantly upregulated genes in DON *vs.* non-DON and DON *vs.* HCs comparisons, respectively. Notably, inflammation-related genes, including *CXCL14, CCL21, HP*, and fibrosis-associated genes such as *MGP, FN1*, and *COL11A1*, were significantly upregulated in DON group. GO enrichment analyses identified immune-related processes like lymphocyte proliferation, cytokine activity, and extracellular matrix remodeling. WGCNA further identified key gene modules associated with inflammation and tissue remodeling in DON, and IRDCGs, such as *CCL21, HP*, and *SLCO2A1*, emerged as the most significant markers. Single-gene GSEA confirmed that these genes are involved in immune response, inflammation, and fibrosis-related processes. Immune cell infiltration analysis using ssGSEA revealed that DON patients exhibited significantly increased infiltration of activated B cells, CD4 T cells, mast cells, and Th1 cells, and correlation analysis showed that IRDGs were significantly associated with multiple immune cell types, particularly activated B cells and regulatory T cells. Finally, qPCR validation of the top 10 IRDEGs in retro-orbital tissues showed that *HP, TPSAB1*, and *PLA2G2A* were significantly upregulated in the DON.

**Conclusions:**

This is the first study to identify the key molecular and immune drivers of DON through bulk transcriptomic analysis, emphasizing the central role of inflammation-related molecules and immune cell infiltration in its pathogenesis. The identified IRDGs and their associated pathways provide novel insights for innovative diagnostic and therapeutic strategies.

## 1 Introduction

Thyroid eye disease (TED) is a significant autoimmune condition observed in 25-30% of individuals with Graves’ disease. This condition presents with a range of symptoms, including proptosis, tearing, photophobia, lid retraction, and diplopia (1, 2). Among its complications, dysthyroid optic neuropathy (DON) is particularly concerning due to its potential to cause severe and rapid vision impairment. Approximately 4-8% of TED patients experience progression to DON, a condition that can drastically impact quality of life (3, 4). However, the diagnostic criteria for DON are largely reliant on clinical signs and symptoms, lacking both sensitivity and specificity. Consequently, most patients recognize the risk only after substantial vision loss has already occurred (5). Current treatment options for DON remain limited, primarily involving high-dose glucocorticoids or orbital decompression.

Despite the pathogenesis of DON remains unclear, the prevailing hypothesis suggests direct compression of the optic nerve by enlarged extraocular muscles (EOMs) at the orbital apex—a mechanism supported by the European Group on Graves’ Orbitopathy (EUGOGO), which found radiological evidence of apical optic nerve compression in 49 out of 56 DON-affected eyes (3). However, the contribution of immune-mediated mechanisms and inflammatory responses to DON pathogenesis has not been fully elucidated. Other contributing factors may include optic nerve stretching from proptosis, increased retrobulbar pressure, inflammation, and altered orbital blood flow, which together could exacerbate optic nerve injury and accelerate visual decline (6, 7).

The core pathogenesis of DON is closely associated with the pathological mechanisms of TED. TED is primarily driven by the aberrant activation and proliferation of orbital fibroblasts, a process influenced by multiple factors. Among the numerous molecular pathways implicated in orbital fibroblast activation, immune and inflammatory dysregulation is recognized as a key driving force, playing a crucial role in both TED progression and the pathogenesis of DON (8, 9). Patients with DON almost always present during the active phase of the disease, characterized by varying degrees of immune cell infiltration within the orbit, including T cells, B cells, monocytes, and mast cells (10, 11). Severe active TED is associated with more significant infiltration by CD4+ T cells and monocytes/macrophages. Clinical Activity Score (CAS) positively correlates with the extent of lymphocyte (total, isolated T cells, isolated B cells) and macrophage infiltration, highlighting the critical link between immune responses, disease activity, and orbital inflammation (11–13). In our previous multicenter, single-blind case-control study, we observed significant immune lymphocyte infiltration and inflammatory cytokine release within the orbits of patients with severe active TED, indicating that immune-mediated inflammation is a key driver of DON pathogenesis (14). The activation of immune responses leads to an increased release of inflammatory and chemotactic factors, exacerbating orbital inflammation (15), and resulting in the aberrant proliferation and activation of central effector cells and target cells, namely orbital fibroblasts, This ultimately leads to extraocular muscle thickening and fat deposition, hallmarks of DON progression (16, 17).

**Figure 1:**
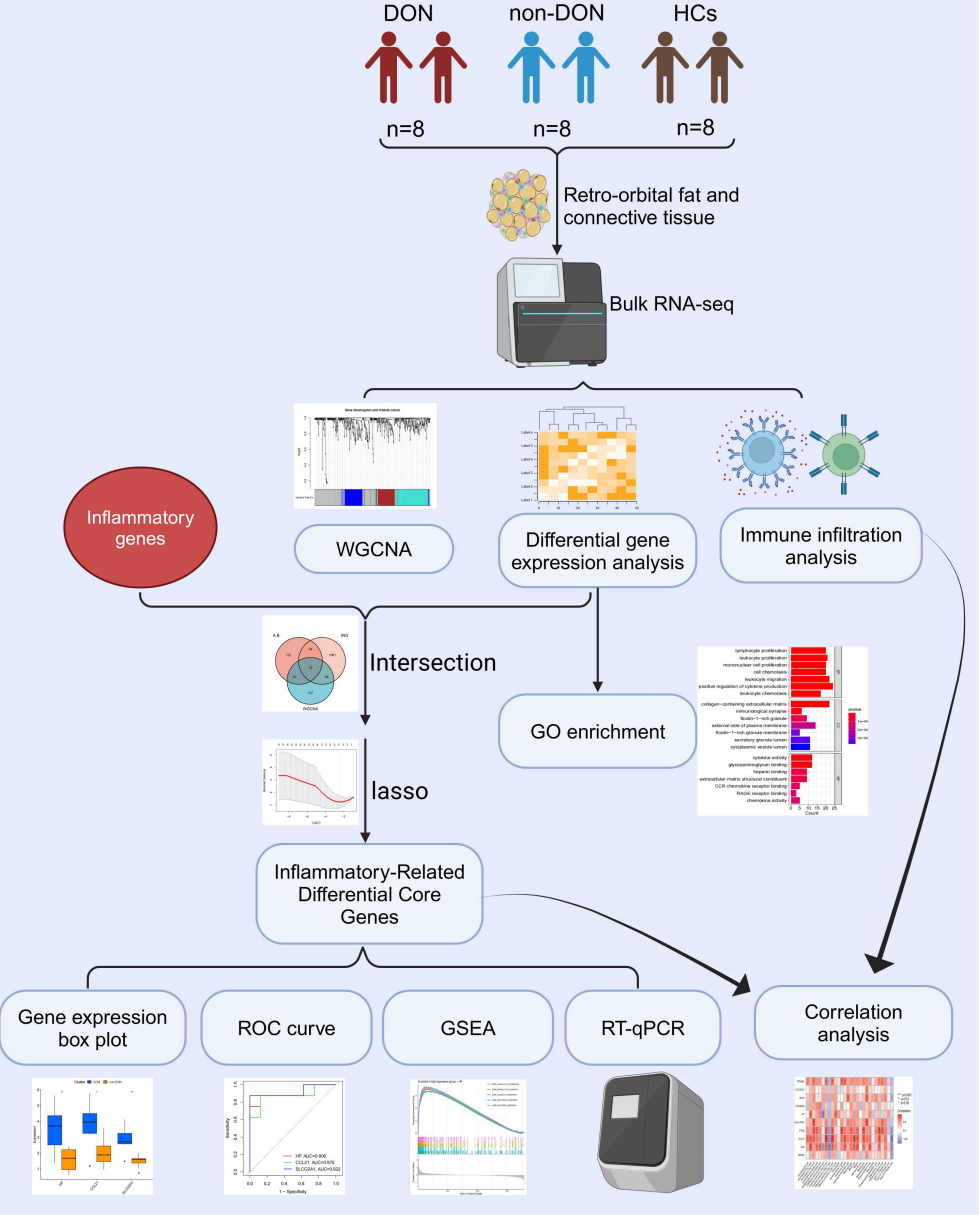
Flow chart of this study. Created with Biorender.

Despite these findings, a detailed understanding of the molecular pathways and inflammation-related genes involved in DON is lacking. To address this research gap, this study employs bulk transcriptomic sequencing on retro-orbital tissue, aiming to reveal the molecular underpinnings of the disease. This approach enables a detailed examination of inflammation-related genes and pathways, potentially uncovering molecular markers for early diagnosis and disease progression. Additionally, given the limited treatment options, identifying novel therapeutic targets through transcriptomic analysis could provide a foundation for developing targeted anti-inflammatory therapies, ultimately enhancing the prognosis and quality of life for DON patients. The study’s methodology flow is detailed in **Figure 1**.

## 2 Methods

### 2.1 Sample collection and ethical approval

In this study, patients with TED were classified into two groups based on the presence or absence of DON: the DON group (DON) and the non-DON group (non-DON), with a healthy control (HC) group also included. Each group consisted of 8 samples. The diagnosis of TED was based on the Bartley criteria (18). The diagnostic criteria for dysthyroid optic neuropathy (DON) required a prior history of TED and at least two of the following clinical manifestations: (1) decreased best-corrected visual acuity (BCVA) < 0.8, (2) abnormal pattern visual evoked potentials (VEP), (3) papilledema, (4) visual field defects, (5) abnormal color vision, and (6) orbital CT or MRI showing orbital apex crowding. The inclusion criteria for healthy controls included: absence of thyroid disease or other autoimmune disorders, and no history of infections, leukemia, anemia, or other relevant diseases. The retro-orbital tissue samples from the DON group were obtained from patients undergoing urgent orbital decompression surgery, while tissue samples from the non-DON group were derived from patients undergoing elective orbital decompression. The retro-orbital fat tissues from the healthy control group were collected from patients undergoing orbital aesthetic surgery. The basic information of the patients is shown in **Table 1**. This study has been reviewed and approved by the Ethics Committee of Shunde Hospital of Southern Medical University (KYLS20240602) and the Ethics Committee of Peking University Third Hospital (M2024362).

**Table 1:** Demographic and clinical data.

	DON (n=8)	non-DON (n=8)	Control (n=8)
Age (year)	54.5 ± 7.81	35 ± 7.74	41.37 ± 20.04
Gender, female N (%)	4 (50.0%)	7 (87.5%)	5 (62.5%)
CAS (point)	3.62 ± 0.74	0.50 ± 0.76	N/A
Duration (year)	0.58 ± 0.24	4.38 ± 3.00	N/A
Orbital irradiation ratio N (%)	0 (0)	0 (0)	N/A
Active ratio N (%)	8 (100)	0 (0)	N/A
Inactive ratio N (%)	0 (0)	8 (100)	N/A
Steroid ratio N (%)	1 (12.5)	1 (12.5)	N/A
Euthyroid ratio N (%)	8 (100)	8 (100)	N/A
Thyrotropin	1.37 ± 1.83	1.61 ± 1.13	N/A
Free Triiodothyronine	4.28 ± 1.59	4.23 ± 1.18	N/A

Data are presented as the average ± standard deviation. CAS, clinical activity score; N, number; N/A, not available; Duration, duration of orbital disease; Steroid ratio, the ratio of intravenous methylprednisolone pulses.

### 2.2 RNA extraction, library preparation, and sequencing analysis

Total RNA was extracted from orbital adipose tissue using Trizol Reagent (Invitrogen Life Technologies) following the manufacturer’s protocol. RNA purity, concentration, and integrity were assessed using a NanoDrop spectrophotometer (Thermo Scientific). For library preparation, mRNA was enriched using poly-T oligo-attached magnetic beads, fragmented, and reverse-transcribed into cDNA. Sequencing libraries were constructed using the Illumina platform with standard protocols (19). The libraries were quantified using a Bioanalyzer 2100 system (Agilent) and sequenced on the NovaSeq 6000 platform (Illumina) to generate paired-end reads. Raw sequencing reads were processed to remove adapter sequences and low-quality reads using Cutadapt (v1.15) (20). Clean reads were aligned to the reference genome using HISAT2 (v2.0.5) (21) for downstream analysis.

### 2.3 Differentially expressed gene analysis and enrichment analysis

We utilized the R package DESeq2 to perform pairwise gene differential analysis among DON, non-DON, and HCs. For comparisons between DON and HCs, as well as between DON and non-DON, we used the criteria of p < 0.05 and |fold change (FC)| > 1. For the comparison between non-DON and HCs, we applied the criteria of p < 0.05 and |fold change (FC)| > 0.5. Additionally, we utilized volcano plots to visualize the differentially expressed genes identified from these comparisons. Further, we employed the R package “clusterProfiler” to perform GO enrichment analyses on these DEGs to elucidate potential molecular mechanisms involved in the pathological processes DON. The results were visualized using bar diagrams. To ensure the reliability of the enrichment outcomes, we applied the Benjamini–Hochberg FDR method to correct the p-values for multiple hypothesis testing, with an FDR < 0.05 indicating statistically significant enrichment.

### 2.4 Weighted gene co-expression network analysis

We utilized WGCNA to identify co-expressed gene modules and investigate their relationship with DON, aiming to pinpoint key genes that play a critical role. To achieve this, we first constructed a weighted adjacency matrix that emphasizes strong gene relationships while penalizing weak correlations, and then transformed it into a Topological Overlap Matrix (TOM) to quantify gene connectivity within the network. The TOM not only measures the connectivity of neighboring genes but also calculates their dissimilarities. To group genes with similar expression profiles, we employed average linkage hierarchical clustering based on the dissimilarities estimated from the TOM, with the resulting gene modules visually represented in a dendrogram, facilitating the identification of module relationships and relevant genes. Finally, we calculated the Pearson correlation coefficients and p-values to assess the association between module genes and TED activity, with the results displayed using heatmaps. We performed GO enrichment analysis on the biological processes of the relevant gene modules.

### 2.4 Identification of inflammatory-related differential core genes

The inflammation-related genes (IRGs) were sourced from the DisGeNET database and the Molecular Signatures Database (MSigDB) (22). A total of 467 IRGs were retrieved from DisGeNET. Additionally, four gene sets, specifically M5932, M15877, M13807, and M38152, were obtained from the MSigDB database for further analysis. We intersected the differentially expressed genes between patients DON and non-DON, the gene set identified by WGCNA that is closely associated with DON, and the inflammatory-related genes, resulting in the identification of 10 Inflammatory-Related Differential Genes (IRDGS). The selected genes were visualized using box plots to illustrate their expression patterns across different groups. We plotted Receiver Operating Characteristic (ROC) curves to assess the accuracy of these genes in distinguishing between DON and non-DON. The area under the curve (AUC) was utilized to evaluate their diagnostic efficacy. We further performed lasso regression analysis using the glmnet R package to identify the three most significant Inflammatory-Related Differential Core Genes (IRDCGs).

### 2.5 Assessment of immune cell infiltration and its correlation with IRDGS

In the expression matrix, the relative infiltration levels of 28 immune cells were quantified using the ssGSEA algorithm. Heatmaps and violin plots were utilized to demonstrate the differences in infiltration levels of these 28 immune cells between two groups. Subsequently, Spearman correlations between these 28 immune infiltrating cells and IRDGS were calculated, followed by visualization using the ‘ggplot2’ package.

### 2.6 Gene set enrichment analysis for single genes

To perform gene set enrichment analysis (GSEA), we categorized the samples into high- and low-expression groups based on the median expression level of the target gene. The GSEA analysis was conducted using the clusterProfiler package, employing the c5.go.Hs.symbols.gmt gene set file to identify significantly enriched biological processes. Log fold change (logFC) values were calculated between the high- and low-expression groups, and pathways with p-values less than 0.05 were considered significantly enriched. For each target gene, the top five significantly upregulated pathways were visualized through enrichment plots for comprehensive analysis.

### 2.7 RT-qPCR analysis

RNA extraction was performed following standard procedures, including tissue homogenization, chloroform extraction, and ethanol precipitation. The quality and concentration of the extracted RNA were assessed using a NanoDrop spectrophotometer. Reverse transcription was carried out with a one-step RT-PCR kit (catalog number: G3337) in a 20 µL reaction system after genomic DNA removal. Quantitative real-time PCR (qPCR) was performed using SYBR Green Master Mix on a real-time PCR system under standard cycling conditions recommended by the manufacturer. Gene expression levels were normalized to the reference gene GAPDH using the 2^-ΔΔCT method. Primer sequences are summarized in **Table 2**. Statistical analysis was performed using the t-test to compare the differences between groups.

**Table 2:** Sequences of primers.

Gene	Primers	Sequence
GAPDH	Forward	GGAAGCTTGTCATCAATGGAAATC
	Reverse	TGATGACCCTTTTGGCTCCC
ACKR1	Forward	ATTCCTTCCCAGATGGAGACTATG
	Reverse	TGCAGAGTCATCCAGCAGGTTA
SELP	Forward	CTTGCCAACCTGTGAGGCTATT
	Reverse	GTCATACTGAAACGCTCTCAAGGA
CCL21	Forward	GCAGCATCTGGACAAGACACC
	Reverse	CTCAGTCCTCTTGCAGCCTTTG
HP	Forward	AGGTTGTTCTACACCCTAACTACTCC
	Reverse	AGGGCTCTTCGGTGTCTTCTT
C4A	Forward	GAAACCCATCTCGTAATAATGTCCC
	Reverse	GAGAGGAGAAGAGCAGGTTGATAC
TPSAB1	Forward	ATCATCGTGCACCCACAGTTCTA
	Reverse	GTTTTCCATTATGGGGACCTTCA
PLA2G2A	Forward	CACTCAGTTATGGCTTCTACGGCT
	Reverse	CAGCAGCCTTATCACACTCACA
HLA-DPA1	Forward	TGATCCAGCGTTCCAACCAC
	Reverse	TGAGGGCACAAAGGTCAGGTAA
CTSG	Forward	GATGTGGAGGGTTCCTGGTG
	Reverse	CTGCAATAACATGATGTCATTCTGG
SLCO2A1	Forward	GCTTTGGGCTCTCCAGTTCT
	Reverse	CTGTGGCACTTACTGGGAGG

## 3 Results

### 3.1 Differentially expressed genes associated with DON and non-DON

**Figure 2:**
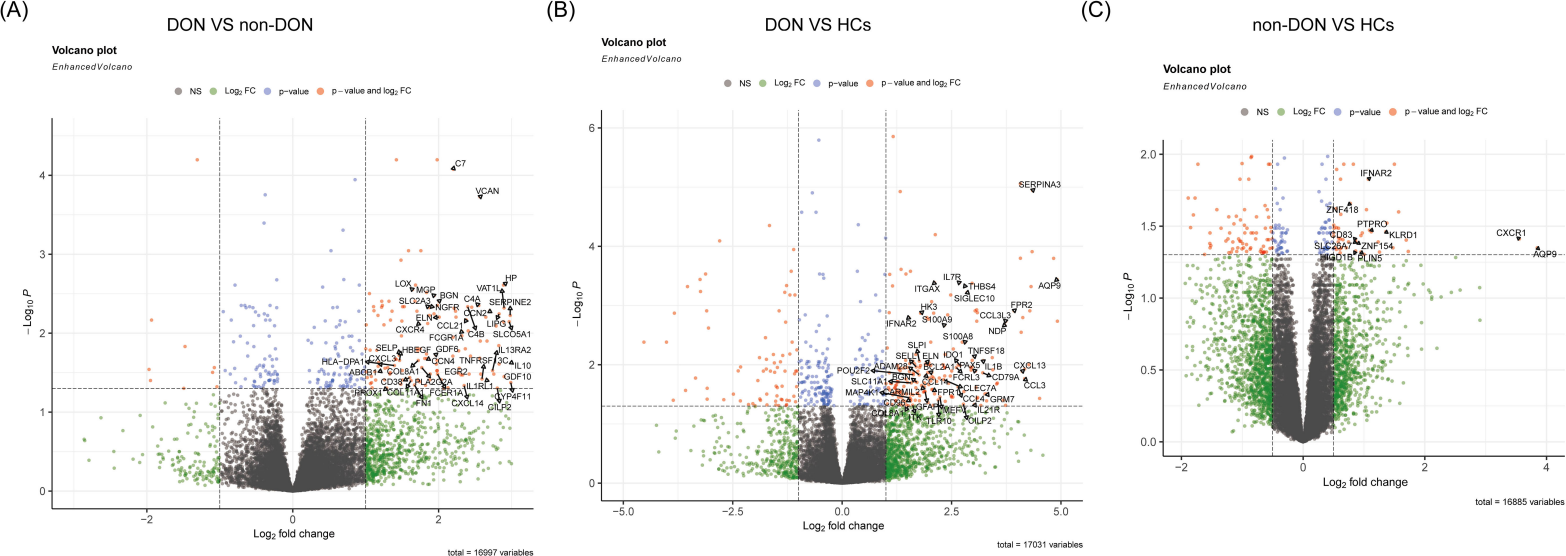
Volcano plots showing differentially expressed genes (DEGs) among DON, non-DON, and healthy controls (HCs). **(A)** Comparison between DON (TED patients with concurrent DON) and non-DON (TED patients without DON), highlighting significantly upregulated genes in the DON group involved in inflammatory and fibrotic pathways. **(B)** Comparison between DON and HCs, identifying DEGs that are markedly upregulated in DON, particularly those associated with immune response and fibrosis. **(C)** Comparison between non-DON and HCs, demonstrating significantly upregulated genes in non-DON compared to healthy controls, though to a lesser extent than in DON. The color coding indicates gene regulation levels: red for genes with significant p-values and high fold changes, blue for significant p-values, and green for high fold changes only.

We conducted pairwise differential gene expression analyses on orbital adipose connective tissue from DON, non-DON, and HCs. We applied the criteria of p_adj < 0.05 and |fold change (FC)| > 1 in the comparisons between DON and non-DON, as well as between DON and HCs, identifying 176 and 202 significantly upregulated genes, respectively. In the comparison between non-DON and HCs, the number of differentially expressed genes was relatively low. Therefore, we adjusted the threshold to |fold change (FC)| > 0.5 to capture potential differences while balancing sensitivity and specificity, resulting in 61 significantly upregulated genes. The results are presented in the volcano plot (**Figures 2A–C**), highlighting the upregulated differentially expressed genes between each group. In the comparison between the DON and non-DON groups, several inflammation-related genes, including CXCL14, CCL21, CXCL3, HP, CXCR4, HLA-DPA1, and SELP, were significantly upregulated in the DON group. Furthermore, fibrosis-associated genes, such as MGP, BGN, FN1, and COL11A1, also showed elevated expression in the DON group. When comparing DON to HCs, a broader set of inflammation-related genes was found to be upregulated, including CCL3, IL7R, CXCL13, IL1B, IL21R, and S100A9. This suggests an intensified immune response in DON. Fibrosis-associated genes, including BGN, COL8A1, and ELN, also showed increased expression, indicating remodeling processes that are likely involved in disease progression.

### 3.2 Enrichment analysis of DEGs

**Figure 3:**
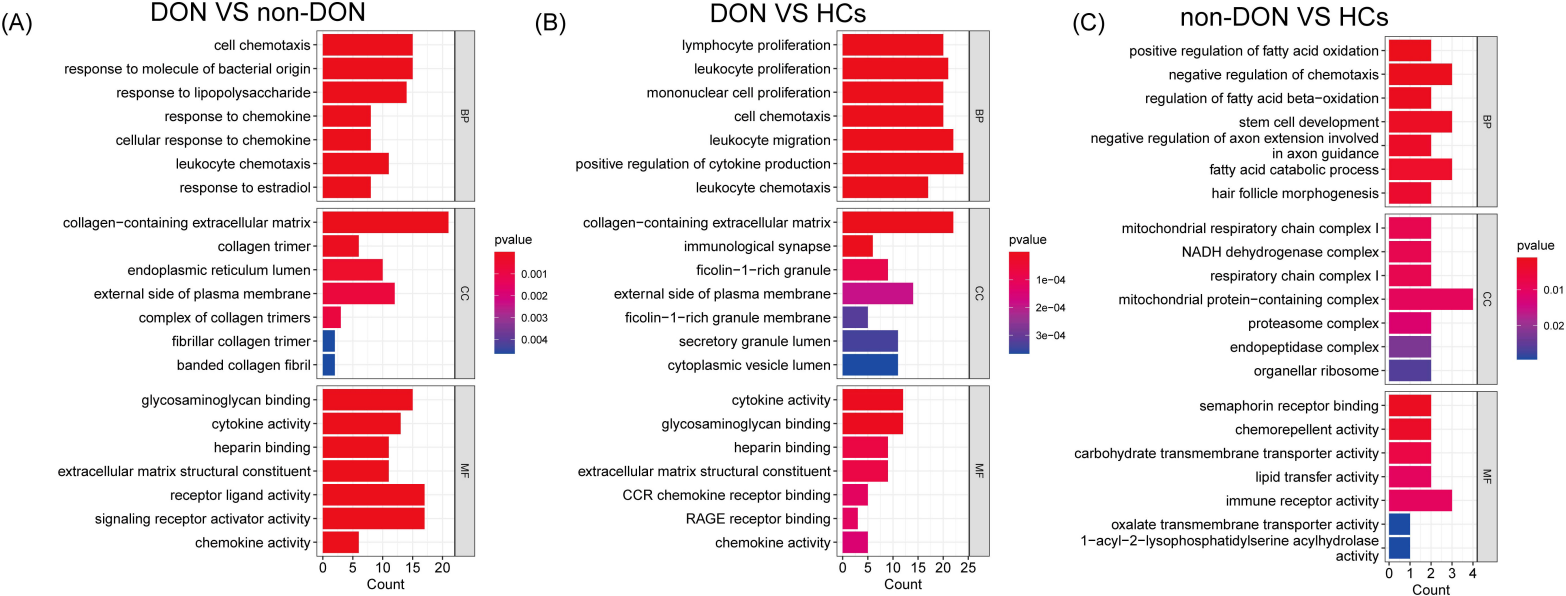
GO enrichment analysis of differentially expressed genes (DEGs) among DON, non-DON, and healthy controls (HCs). **(A)** In the comparison between DON and non-DON, Biological Processes (BP) are enriched in immune and inflammatory responses, Cellular Components (CC) in collagen matrix, and Molecular Functions (MF) in cytokine activity. **(B)** In the comparison between DON and HCs, BP terms are enriched in immune processes, CC in collagen matrix and immunological synapse, and MF in cytokine activity. **(C)** In the comparison between non-DON and HCs, BP terms are enriched in fatty acid oxidation, CC in mitochondrial complexes, and MF in Sema receptor binding. Color indicates p-value significance.

To investigate the biological functions of differentially expressed genes (DEGs) between DON and non-DON patients, we conducted Gene Ontology (GO) enrichment analysis, categorized into Biological Process (BP), Cellular Component (CC), and Molecular Function (MF) (**Figure 3**). Compared to non-DON, DON patients showed significant enrichment in BP terms related to immune and inflammatory responses, including cell chemotaxis, response to bacterial molecules, lipopolysaccharide, and chemokines. In CC, DEGs were enriched in structural components such as collagen-containing extracellular matrix, collagen trimer, endoplasmic reticulum lumen, and plasma membrane. For MF, enriched terms included glycosaminoglycan binding, cytokine activity, heparin binding, extracellular matrix constituents, and chemokine activity. When comparing DON to HCs, DEGs in BP were significantly enriched in immune-related processes like lymphocyte proliferation, leukocyte migration, and cytokine production. CC enrichments included collagen matrix, immunological synapse, and plasma membrane components, while MF terms showed enrichment in cytokine activity, glycosaminoglycan binding, CCR chemokine receptor binding, and RAGE receptor binding. These findings underscore the enhanced immune activity, extracellular matrix remodeling, and signaling in active TED, suggesting key pathways involved in TED pathology and potential therapeutic targets.

### 3.3 Construction of co-expression network and identification of DON core genes

**Figure 4:**
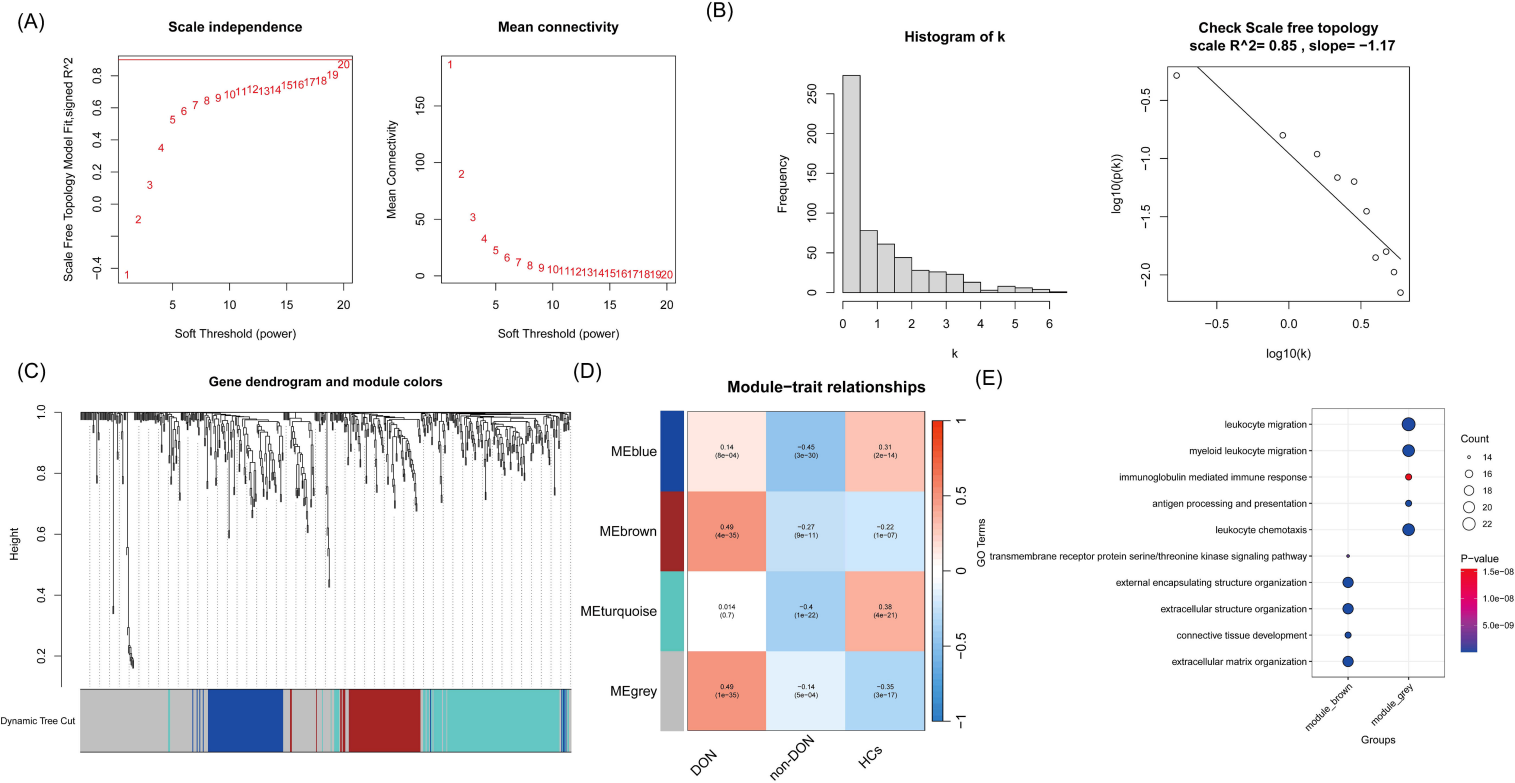
**(A)** Relationship between soft threshold (power) and the scale-free topology model fit and average connectivity. The left panel shows the scale-free topology model fit (R² value) at different soft threshold powers, while the right panel shows the average connectivity at different soft threshold powers. As the soft threshold increases, the average connectivity gradually decreases. **(B)** Network connectivity distribution and validation of scale-free property. The left panel displays a histogram of connectivity (k), showing that most nodes have low connectivity, which is consistent with the characteristics of a scale-free network. The right panel shows a linear fit with an R² value of 0.85, indicating that the network topology approximates a scale-free distribution. **(C)** Gene clustering dendrogram and module division. The dendrogram shows the clustering relationships between genes, with genes assigned to different modules using the dynamic tree-cutting method. **(D)** Correlation analysis between modules and phenotypes. The plot shows the correlation coefficients and p-values between different gene co-expression modules and various phenotypes (DON, non-DON, HCs). The color intensity reflects the strength of the correlation, with red indicating positive correlations and blue indicating negative correlations. **(E)** GO enrichment analysis of the grey and brown module genes, with a focus on biological processes (BP terms), is visualized using a bubble plot.

To more accurately identify hub genes associated with DON, we constructed a gene co-expression network using the WGCNA algorithm. The soft threshold was set to 20 to satisfy the scale-free topology criterion, with a corresponding R² value of 0.85, resulting in a network with high average connectivity (**Figures 4A, B**). A hierarchical clustering dendrogram based on gene correlation was generated, leading to the identification of four distinct gene modules (**Figure 4C**). We then calculated the correlation between the module eigengenes and the DON phenotype, ultimately identifying the “grey” module as the most clinically relevant in DON (p = 1 × 10^-^³^5^) (**Figure 4D**). Further GO enrichment analysis of two gene modules strongly associated with DON, focusing on biological processes (BP terms), revealed distinct functional characteristics (**Figure 4E**). The grey module was significantly enriched in inflammation-related processes, such as leukocyte migration and myeloid leukocyte migration, suggesting a critical role in recruiting and localizing immune cells at sites of inflammation. Additionally, immunoglobulin-mediated immune response and antigen processing and presentation were prominently enriched, indicating the involvement of this module in humoral immunity and adaptive immune functions. In contrast, the brown module was primarily enriched in processes related to tissue and cellular structure, including extracellular structure organization and extracellular matrix organization, which are closely associated with matrix protein regulation and tissue remodeling. Furthermore, connective tissue development was significantly enriched, highlighting the potential role of this module in maintaining and repairing tissue structure.

### 3.4 Identification of inflammatory-related differential core genes

**Figure 5:**
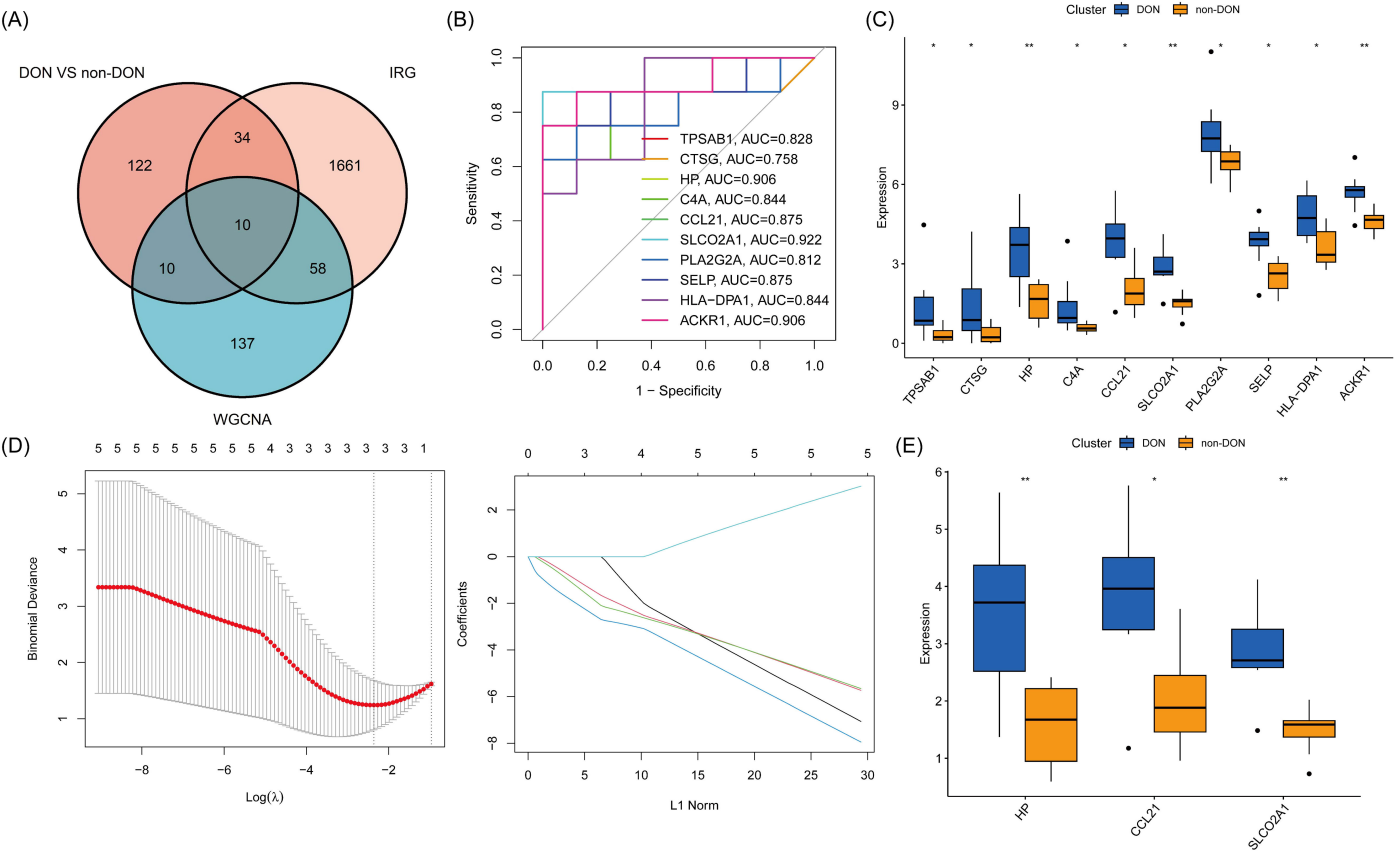
**(A)** Venn diagram illustrating the overlap of differentially expressed genes (DEGs) between DON and non-DON groups, inflammation-related genes (IRGs), and genes identified by WGCNA analysis. **(B)** ROC curves for 10 genes, illustrating their diagnostic performance in distinguishing DON from non-DON groups. The plot shows sensitivity versus 1-specificity for each gene, with the area under the curve (AUC) values indicating the diagnostic accuracy. **(C)** Box plot showing expression levels of 10 core genes in DON and non-DON groups. **(D)** IRDCGS were selected by the LASSO logistic regression algorithm with penalized parameter adjustment by 10-fold cross-validation. **(E)** Box plot showing expression levels of IRDCGS in DON and non-DON groups. Significant differences in gene expression between the two groups are indicated by * (p < 0.05) and ** (p < 0.01).

To investigate the role of inflammation-related genes in the pathogenesis of DON, we intersected the DEGs from the DON and non-DON groups with genes from the gray module and known inflammation-related genes, resulting in a total of 10 Inflammatory-Related Differential Genes (IRDGS) (**Figure 5A**). In the subsequent ROC curve analysis, we assessed the sensitivity and specificity of these genes in distinguishing between DON and non-DON patients by examining their area under the curve (AUC) values. Genes with higher AUC values, such as SLCO2A1 (AUC=0.922), CCL21 (AUC=0.875), HP (AUC=0.906), and ACKR1 (AUC=0.906), demonstrated a stronger potential for differentiating DON from non-DON patients, underscoring their significance in the pathogenesis of DON (**Figure 5B**). Additionally, box plots were used to visualize the expression differences of these genes between the DON and non-DON groups (**Figure 5C**). Lasso regression analysis further identified three key Inflammatory-Related Differential Core Genes (IRDCGs), namely CCL21, SLCO2A1, and HP (**Figures 5D, E**).

### 3.5 Functional enrichment analysis of IRDCGS using single-gene GSEA

**Figure 6:**
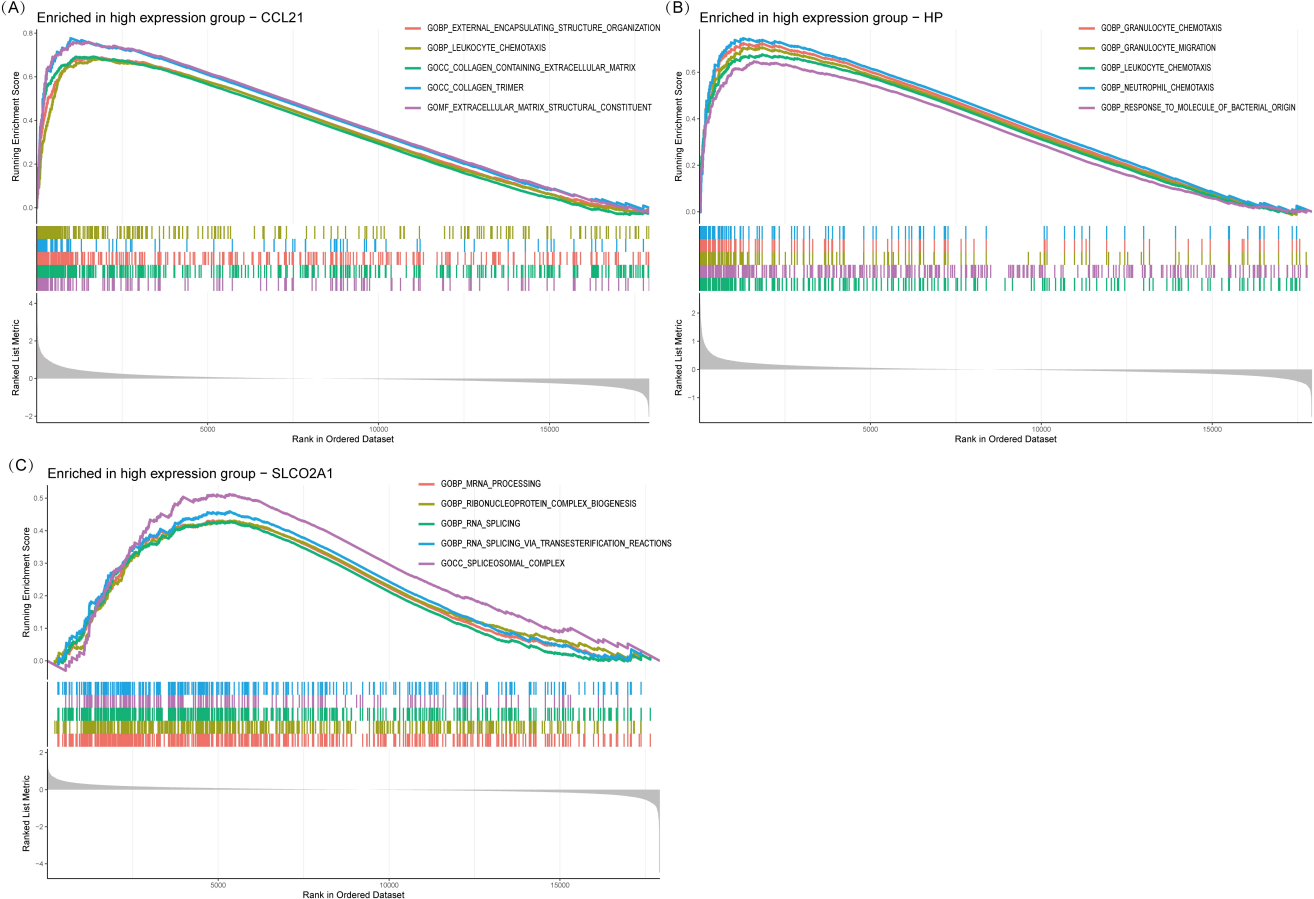
Single gene GSEA. **(A)** GSEA results of CCL21. **(B)** GSEA results of HP. **(C)** GSEA results of SLCO2A1.

Single-gene GSEA analysis revealed that the three IRDCGS, namely CCL21, HP, and SLCO2A1, are closely associated with multiple significant biological processes (**Figures 6A–C**). CCL21 was significantly enriched in inflammation- and fibrosis-related biological processes, including GOBP_LEUKOCYTE_CHEMOTAXIS (leukocyte chemotaxis) and GOCC_COLLAGEN_CONTAINING_EXTRACELLULAR_MATRIX (collagen-containing extracellular matrix), highlighting its dual role in immune cell recruitment and fibrotic progression. HP was significantly enriched in biological processes associated with immune cell migration and inflammation, such as GOBP_GRANULOCYTE_CHEMOTAXIS (granulocyte chemotaxis), GOBP_GRANULOCYTE_MIGRATION (granulocyte migration), GOBP_LEUKOCYTE_CHEMOTAXIS (leukocyte chemotaxis), and GOBP_NEUTROPHIL_MIGRATION (neutrophil migration), underscoring its role in promoting immune cell recruitment and amplifying local inflammatory responses. SLCO2A1 was significantly enriched in RNA regulatory processes, including GOBP_MRNA_PROCESSING (mRNA processing), GOBP_RNA_SPLICING (RNA splicing), and GOBP_RNA_SPLICING_VIA_TRANSESTERIFICATION_REACTION (RNA splicing via transesterification reaction), suggesting its potential involvement in transcriptional and post-transcriptional regulation of gene expression, which may influence the progression of DON.

### 3.6 Immune cell infiltration and its correlation with inflammatory-related differential core genes

**Figure 7:**
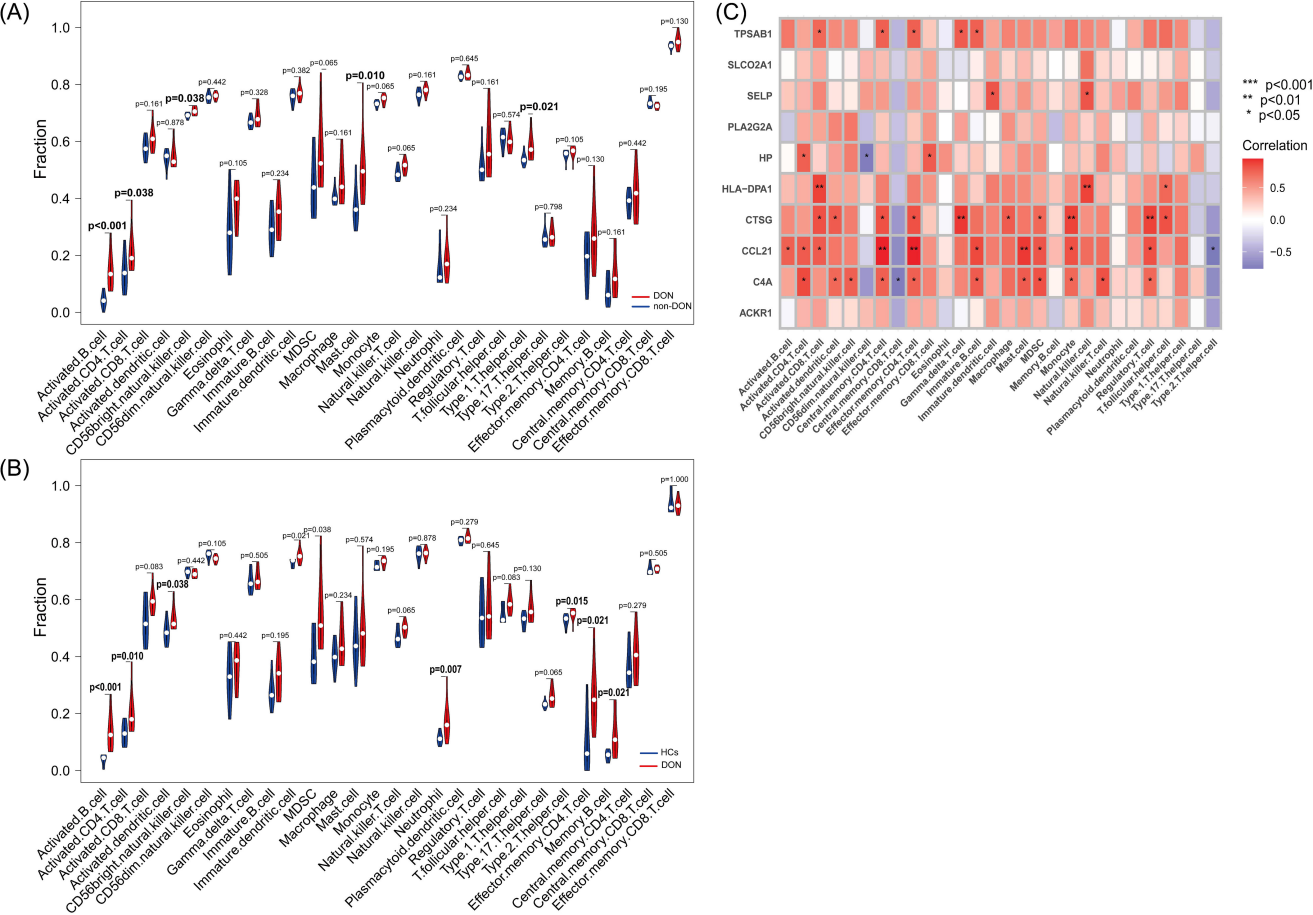
**(A)** Differences in immune infiltration between DON and non-DON based on the ssGSEA algorithm using violin plots. **(B)** Differences in immune infiltration between DON and HCs based on the ssGSEA algorithm using violin plots. **(C)** Pearson correlation analysis showing the correlation of IRDGs with infiltrated immune cells.

We conducted ssGSEA to analyze differences in immune cell infiltration among DON patients, non-DON patients, and healthy controls, with results visualized using violin plots. In the comparison between DON and non-DON TED patients (**Figure 7A**), significant increases were observed in several immune cell types within the DON group. Specifically, activated B cells and activated CD4 T cells showed markedly higher infiltration levels in DON patients, with p-values of <0.001 and 0.038, respectively. Additionally, DON patients exhibited elevated levels of mast cells (p=0.010), CD56 bright natural killer cells (p=0.038), and Type 1 T helper cells (p=0.021), suggesting that these immune cell types may be central to the inflammatory processes characteristic of DON.

Similarly, comparisons between DON patients and healthy controls (HCs) (**Figure 7B**) revealed significant immune cell infiltration differences. DON patients had significantly higher levels of activated B cells (p < 0.001) and activated CD4 T cells (p=0.010) compared to HCs, indicating a heightened immune response associated with the disease. Additional immune cell types, including activated dendritic cells (p=0.038), neutrophils (p=0.007), effector memory CD4 T cells (p=0.021), and memory B cells (p=0.021), were also significantly more abundant in DON patients, suggesting their involvement in the immune-mediated pathology of DON.

The correlation heatmap (**Figure 7C**) illustrates the association between IRDGs and immune cell infiltration in the DON group. Each cell represents the correlation between a specific IRDG and an immune cell type, with the color intensity indicating the correlation strength. Positive correlations are represented in shades of red, while negative correlations are shown in blue. Notably, several IRDCGs, such as CCL21, showed significant positive correlations with multiple immune cell types, particularly activated B cells, central memory CD4 T cells, and regulatory T cells. This suggests that these genes may play a role in promoting immune cell infiltration and activation in the orbital tissues of DON patients. The observed correlations highlight the complex interactions between these genes and various immune cell subsets, suggesting that specific IRDGs could drive immune cell recruitment and inflammation in DON.

### 3.7 The expression levels of the DE-IRGs

**Figure 8:**
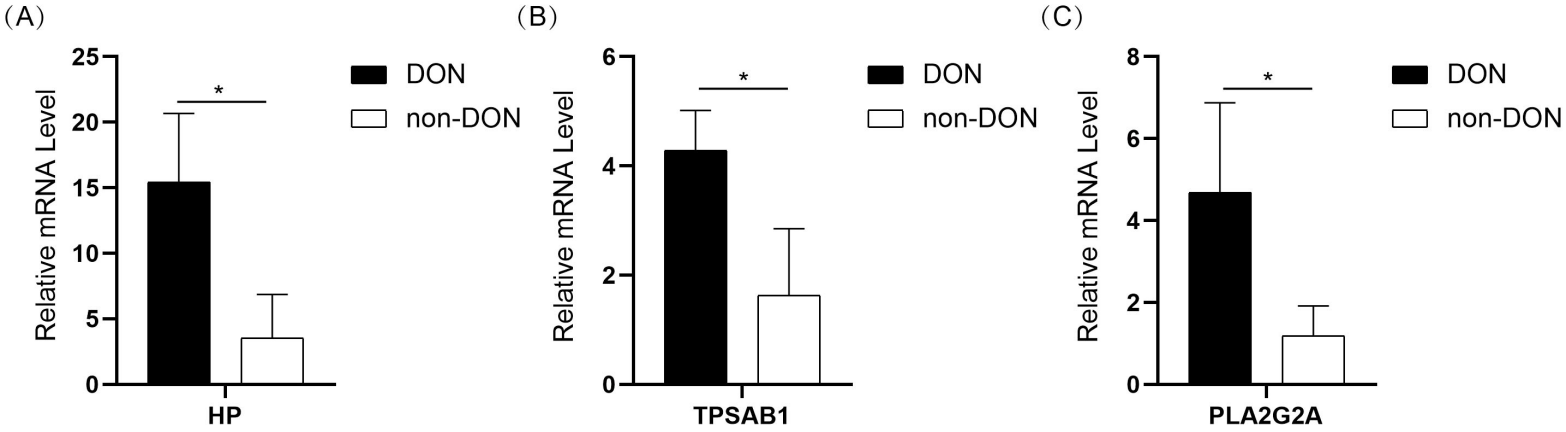
Bar plot showing the expression levels of HP **(A)**, TPSAB1 **(B)**, and PLA2G2A **(C)** in the DON and non-DON groups. Significant differences in gene expression between the two groups are indicated by * (p < 0.05).

To further validate the expression of 10 IRDEGs in the retro-orbital tissues of DON and non-DON patients, RNA was extracted from the retro-orbital tissues and analyzed via qPCR. The results demonstrated the expression levels of HP, TPSAB1, and PLA2G2A were significantly upregulated in the DON group (**Figures 8A–C**). Although other genes exhibited an upward trend in expression in the DON group, the differences were not statistically significant (**Supplementary Figure 1**).

## 4 Discussion

To the best of our knowledge, this is the first study to investigate the molecular and immune mechanisms of DON using a bulk transcriptomic approach in TED orbital connective tissue. This was followed by analysis with various computational algorithms and validation through quantitative real-time PCR. Our findings revealed significant differences in the retro-orbital immune microenvironment of DON patients. By integrating differential gene expression analysis, co-expression network construction, functional enrichment studies, and immune cell infiltration analysis, we identified key pathways and inflammation-related core genes associated with DON.

The precise pathogenesis of DON remains incompletely understood. Previous studies, including those conducted by our team, have shown distinct patterns of immune cell infiltration and inflammatory molecule expression in the retro-orbital tissues of TED patients (14, 23). These immune components interact extensively with orbital fibroblasts (OFs) (24), leading to their proliferation, activation, and differentiation. This process results in the deposition of collagen and glycosaminoglycans, increasing the volume of extraocular muscles and orbital fat/connective tissues (25). The subsequent crowding of orbital contents exerts pressure on the optic nerve, ultimately leading to the development of DON.

The autoimmune-mediated inflammatory process is a critical factor driving the progression of TED to DON, particularly through the pathological processes of immune cell infiltration and tissue remodeling within the retro-orbital space (26). Retro-orbital tissues in TED patients exhibit significant immune cell infiltration, including CD4+ T cells, B cells, macrophages, and mast cells (27–29). These immune cells release a range of inflammatory cytokines, chemokines, and growth factors, further intensifying the local inflammatory environment (30). This exacerbated inflammatory process leads to tissue edema and orbital congestion, ultimately resulting in optic nerve compression at the orbital apex (31–33). This compression, combined with the expansion of extracellular matrix and fibrotic changes, further impairs the blood supply to the optic nerve, leading to optic nerve dysfunction and progressive vision loss.

This study utilized bulk RNA sequencing to explore the molecular and immune mechanisms of DON, revealing significant differences in the retro-orbital immune microenvironment of DON patients. Through a comprehensive approach combining differential gene expression analysis, co-expression network construction, functional enrichment studies, and immune cell infiltration analysis, we identified key pathways and inflammatory-related core genes associated with DON.

Differential gene expression and pathway enrichment analysis revealed that several inflammation- and fibrosis-related genes (e.g., CXCL14, CCL21, HP, MGP, BGN) were significantly upregulated in DON patients. These gene expression changes suggest an enhanced inflammatory state and extracellular matrix remodeling process in DON. Functional enrichment analysis showed that DON-related genes were significantly enriched in immune response pathways (such as leukocyte migration and cytokine production) and structural pathways (such as collagen-containing extracellular matrix).

By integrating differential gene expression data, co-expression networks, and known inflammation-related genes, we identified 10 inflammatory-related differential genes (IRDGs), among which CCL21, HP, and SLCO2A1 demonstrated the strongest associations with DON based on Lasso regression analysis. Single-gene GSEA analysis revealed the following: CCL21 was enriched in pathways related to leukocyte chemotaxis and collagen-containing extracellular matrix, suggesting its dual role in immune cell recruitment and fibrotic progression. HP was enriched in pathways related to granulocyte and neutrophil migration, highlighting its role in enhancing immune cell recruitment and amplifying local inflammatory responses. SLCO2A1 was associated with RNA processing and splicing, indicating its potential involvement in transcriptional and post-transcriptional regulation of gene expression, which may promote the progression of DON.

The effectiveness of hormonal therapy in the treatment of DON highlights the significant contribution of immune cells to the progression of the disease (34, 35). Previous studies have shown a marked increase in T cells and B cells infiltration in the eye tissues of patients with severe active disease (36). Additionally, the abnormal aggregation of mast cells has been reported to promote tissue fibrosis and inflammatory responses in TED, which aligns with the findings of our study (29, 37). In this study, ssGSEA analysis revealed significant differences in immune cell infiltration between DON patients, non-DON patients, and healthy controls (HCs). In DON patients, the levels of activated B cells, CD4 T cells, mast cells, and Th1 cells were significantly elevated, reflecting the prominent immune activation characteristic of the disease. Additionally, compared to HCs, DON patients exhibited significantly higher levels of activated dendritic cells, neutrophils, and memory B cells, suggesting interactions between innate and adaptive immunity during the pathogenesis of DON. Correlation analysis revealed that inflammatory-related differential core genes (IRDCGs) such as CCL21 were significantly positively correlated with activated B cells, central memory CD4 T cells, and regulatory T cells. These findings indicate that these genes play a crucial role in promoting immune cell infiltration and activation, further supporting their significance in the progression of DON.

CCL21 plays a critical role in various immune-related diseases. The CCL21-CCR7 axis exacerbates the immune-inflammatory response in dry eye disease (DED) by promoting the migration of dendritic cells to lymphatic vessels, thereby amplifying the inflammatory process (38). CCL21 also regulates the migration of dendritic cells and T cells through its interaction with CCR7. Its C-terminal tail influences the binding of glycosaminoglycans (GAGs) and chemotactic effects, which are essential for antigen presentation and immune regulation in chronic inflammatory diseases (39). Additionally, CCL21 enhances T cell infiltration and amplifies immune inflammation in Th1-type immune responses in various autoimmune diseases. In inflammatory bowel disease (IBD), CCL21 drives gut inflammation by promoting the activation of dendritic cells and T cells via CCR7 (40). CCL21 may influence the retro-orbital immune microenvironment through immune cell recruitment, thus contributing to the progression of TED to DON. Haptoglobin (HP) is a plasma glycoprotein (41) that plays a role in regulating immune responses and inflammation. HP modulates tissue repair processes by regulating the function of monocytes and macrophages (42). It is closely associated with the pathogenesis of various inflammatory diseases, such as atherosclerosis and chronic inflammation related to diabetes (43). In inflammatory diseases, HP levels are positively correlated with disease activity, making it a useful biomarker for inflammation (44). In this study, HP showed diagnostic significance in distinguishing different stages or activity levels of TED, particularly in identifying patients at risk for DON. Prostaglandin E2 (PGE2) exacerbates local inflammation by driving a pro-inflammatory phenotype in orbital fibroblasts. PGE2 significantly enhances IL-6 expression, with a more pronounced effect in orbital fibroblasts compared to dermal fibroblasts. This process is mediated through the EP2 receptor and involves pre-transcriptional upregulation of IL-6, increased gene promoter activity, and enhanced CREB phosphorylation, which promotes its nuclear localization and binding to target DNA sequences (45). In this study, we found that the significantly upregulated SLCO2A1 gene in DON patients may further amplify this mechanism. As a PGE2 transporter, SLCO2A1 plays a crucial role in the uptake and clearance of PGE2. Its elevated expression may regulate the effective concentration of PGE2 in orbital tissues, thereby influencing local inflammation and the progression of the disease (46, 47). TPSAB1 (Tryptase Alpha/Beta 1) is a key effector in mast cell degranulation. Tryptase can degrade extracellular matrix (ECM) proteins, activate matrix metalloproteinases (MMPs), and release pro-inflammatory cytokines, thereby exacerbating tissue inflammation and remodeling (48). Previous studies have indicated that mast cells and their products are involved in the activation of orbital fibroblasts (29), promoting the generation of prostaglandin E2 (PGE2) and hyaluronan. This process is mediated by IL-4 secreted by mast cells, which upregulates the expression of cyclooxygenase-2 (COX-2), further enhancing prostaglandin synthesis and aggravating inflammation (49). Our study suggests that the increased infiltration of mast cells in the retro-orbital region of DON patients, along with the significant upregulation of TPSAB1 transcription in this area, may reflect enhanced mast cell activity and its contribution to fibroblast activation. PLA2G2A (Phospholipase A2 Group IIA) hydrolyzes phospholipids to generate arachidonic acid, which drives the synthesis of prostaglandins (e.g., PGE2) and leukotrienes. These pro-inflammatory mediators further amplify the local inflammatory response, leading to tissue edema and immune cell infiltration (50). Additionally, PLA2G2A has been shown to be closely associated with fibrosis, and its elevated expression in other inflammatory diseases, such as ulcerative colitis and atherosclerosis, correlates with disease activity and tissue damage (51, 52).

CCL21, HP, and SLCO2A1 have been identified as key biomarkers in DON, holding significant potential for clinical translation. In early diagnosis, these biomarkers could serve as molecular indicators, enabling the detection of DON before severe vision impairment occurs. This could help overcome the limitations of current diagnostic criteria, which rely primarily on clinical symptoms with limited sensitivity and specificity (32). Furthermore, these biomarkers may aid in disease stratification, allowing for the differentiation of distinct DON subtypes. CCL21 may reflect the degree of immune cell infiltration, HP, as an acute-phase protein, could indicate systemic inflammatory activity, while SLCO2A1 may be involved in prostaglandin transport and tissue remodeling (40, 41, 46). In personalized treatment, these biomarkers could provide valuable insights for precision therapy. For instance, patients with high CCL21 expression might benefit from immune-modulating therapies, whereas those with elevated HP levels may respond more effectively to systemic anti-inflammatory treatment. Additionally, dynamic monitoring of these biomarkers could be instrumental in predicting disease progression and assessing treatment response. Future research should focus on validating these findings in larger patient cohorts.

Our study holds potential value for the diagnosis and treatment of DON. Several inflammation-related genes, such as HP, show promise as biomarkers for the early detection of DON. Furthermore, the enrichment of inflammation and fibrosis-related pathways suggests potential therapeutic targets. For instance, targeting CCL21-mediated chemotaxis or HP-driven granulocyte recruitment may help alleviate inflammation and tissue damage. Future research should focus on validating these findings in larger patient cohorts and exploring the therapeutic effects of targeting these pathways. Additionally, integrating multi-omics data (such as proteomics and metabolomics) may provide further insights into the pathophysiology of DON.

Although our study has provided useful information regarding the mechanisms of TED, it is not without limitations. First, due to the constraints of clinical sample availability, each group consisted of only eight samples, with some degree of imbalance. Second, functional validation experiments are currently lacking. Future studies should focus on further validating key genes through knockdown or overexpression experiments in orbital fibroblasts or immune cells. Lastly, while this study primarily elucidates molecular expression differences and immune cell infiltration at the transcriptomic level, further molecular validation is necessary. Future research should incorporate experimental approaches such as Western blotting and immunohistochemistry to confirm protein-level differences and immune cell infiltration patterns. Moreover, isolating immune cells from retro-orbital tissues will be essential to assess their activation markers and cytokine expression. Furthermore, integrating immune cell infiltration phenotypes with clinical data from patients will provide deeper insights into the relationship between immune responses and disease progression, enhancing the clinical relevance of these findings.

## 5 Conclusion

In conclusion, this study identified key molecular and immune drivers of DON, highlighting the central role of inflammation-related molecules and immune cell infiltration in its pathogenesis. The identified IRDGs and their associated pathways offer novel insights for innovative diagnostic and therapeutic strategies. These findings not only advance our understanding of DON but also provide valuable perspectives for early diagnosis and improving the clinical prognosis of this severe complication of TED.

## Data Availability

The data presented in this study are deposited in the National Center for Biotechnology Information (NCBI) Sequence Read Archive (SRA), accession number PRJNA1229840.
