# Supplementary figures and images for: Inflammatory profiling and immune cell infiltration in dysthyroid optic neuropathy: insights from bulk RNA sequencing
Source: Front Immunol. 2025 Mar 12;16:1550694. doi: 10.3389/fimmu.2025.1550694 (PMC11951427; doi:10.3389/fimmu.2025.1550694)

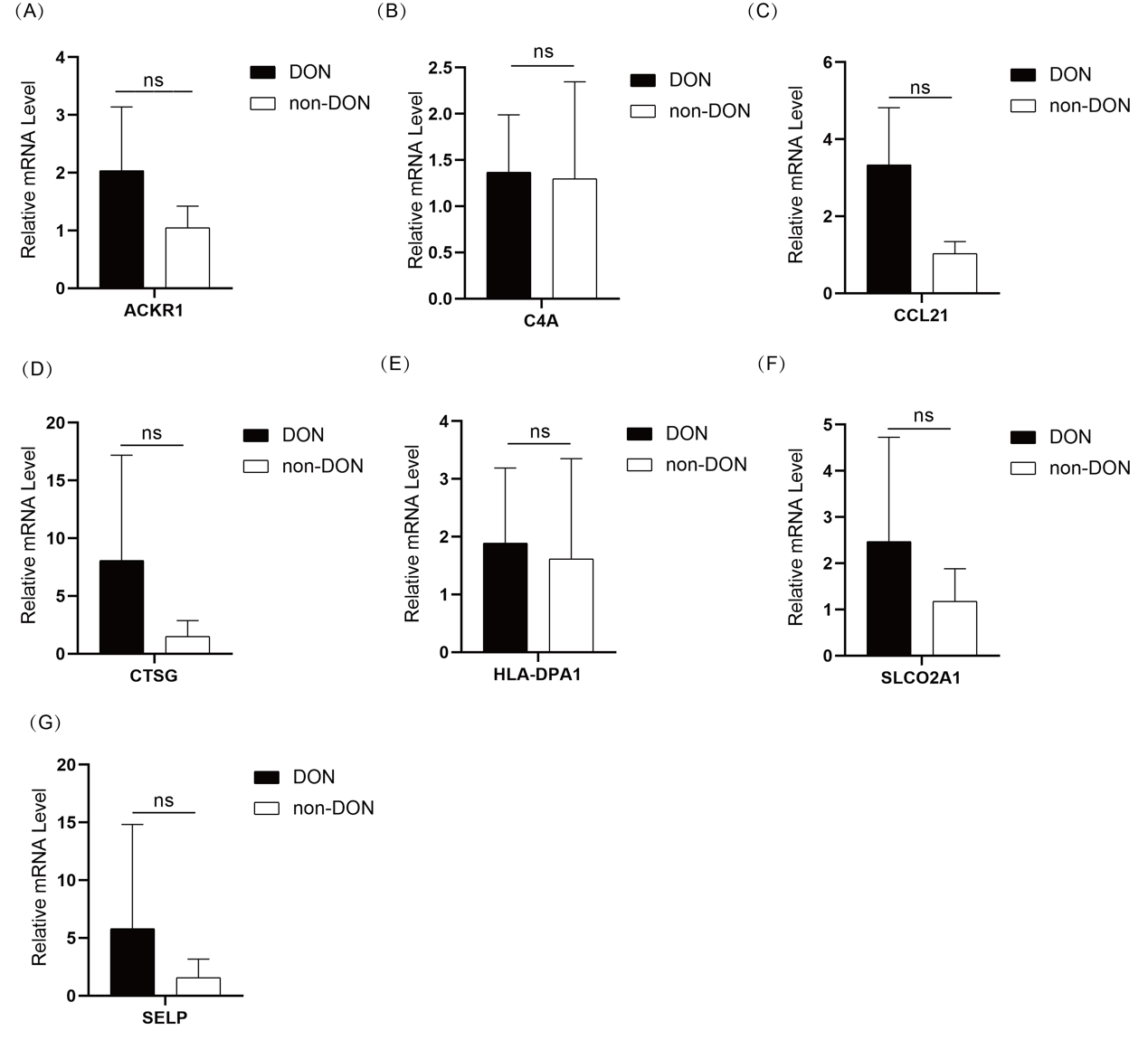


Supplementary figure 1

Supplement: Supplementary file 1 [file Supplementaryfile1.docx]
